# SnRNA-Seq analysis reveals ten hub genes associated with alveolar epithelial cell injury during pulmonary acute respiratory distress syndrome

**DOI:** 10.1016/j.heliyon.2023.e17160

**Published:** 2023-06-12

**Authors:** Haoran Chen, Jinqiu Ding, Haoyue Xue, Xinyi Tang, Yao Yan, Yongpeng Xie

**Affiliations:** a Kangda College of Nanjing Medical University, Zip Code 222000, Lianyungang City, Jiangsu Province, China; b The Institute of Emergency Medicine of Lianyungang, 222000, Lianyungang City, Jiangsu Province, China; c Department of Emergency and Critical Care Medicine, Lianyungang Clinical College of Xuzhou Medical University, No. 6 Zhenhua Road, Zip Code 222000, Lianyungang City, Jiangsu Province, China; d Department of Emergency and Critical Care Medicine, Lianyungang Clinical College of Nanjing Medical University, No. 6 Zhenhua Road, Zip Code 222000, Lianyungang City, Jiangsu Province, China; e Department of Emergency Medicine, Lianyungang Clinical College of Nanjing Medical University, The First People’s Hospital of Lianyungang City, Zip Code 222000, Lianyungang City, Jiangsu Province, China; f Department of Critical Care Medicine, The Second People’s Hospital of Lianyungang City, Zip Code 222000, Lianyungang City, Jiangsu Province, China

**Keywords:** Pulmonary acute respiratory distress syndrome, Type 2 alveolar epithelial cells, SnRNA-Seq

## Abstract

**Background:**

Alveolar epithelial cell injury is a key factor in the occurrence and development of pulmonary acute respiratory distress syndrome (ARDSp). Yet the gene expression profile of alveolar epithelial cells of patients with ARDSp remains unclear.

**Methods:**

We analyzed single nuclear RNA sequencing (snRNA-Seq) data from autopsy lung tissues of both ARDSp patients and healthy donors. Sequence data for type 2 alveolar epithelizal cells (AT2) were extracted by the Seurat package. Differentially expressed genes (DEGs) in AT2 were identified by the criteria |log2FC| ≥ 0.25 and *P* < 0.05 with DESeq2. A protein interaction network was constructed using Search Tool for the Retrieval of Interacting Genes (STRING) and Cytoscape software to identify hub genes. We then constructed an ARDSp rat model through induction by lipopolysaccharide (LPS) airway instillation. Left lung RNA was extracted and sequenced via Illumina Hiseq platforms. Analysis of the rat RNA sequencing data was then used to verify hub genes. Gene ontology (GO) and Kyoto Encyclopedia of Genes and Genomes (KEGG) analyses were performed on the identified hub genes.

**Results:**

In AT2, a total of 289 genes were identified as differentially expressed between those from ARDSp patients and healthy donors, and these included 190 upregulated and 99 downregulated genes. Ten hub genes were further identified (*RPS27A, ACTG1, CAV1, HSP90AA1, HSPA5, CCND1, ITGA3, B2M, NEDD4L*, and *SEMA5A*). There was a similar expression trend of *HSPA5* between rat RNA and snRNA sequencing data.

**Discussion:**

ARDSp altered the gene expression profile of AT2. The identified hub genes were enriched in biological processes mainly involved in cell growth and transformation. Relatedly, ferroptosis and autophagy are possibly involved in AT2 injury during ARDSp. These novel insights into ARDSp may aid the discovery of potential targets for the diagnosis and treatment of ARDSp.

## 1 Introduction

Acute respiratory distress syndrome (ARDS) is a common disease in critical medicine, with an intensive care unit (ICU) incidence rate of about 10% [1]. ARDS has both a high incidence and mortality, which exacerbates its health burden on society.

Pulmonary acute respiratory distress syndrome (ARDSp), a subtype of ARDS, is caused by pneumonia, pulmonary contusion, drowning, and inhalation of toxic substances [2]. Its initial injuries mainly occur in alveolar epithelial cells [3]. Type 2 alveolar epithelial cells (AT2) cells contribute to the occurrence and development of ARDSp as they constitute 2–5% of the total alveolar surface area [4] and two-thirds of alveolar surface cells [5].

They maintain homeostasis in the lungs through the secretion of surfactants, regeneration, and regulation of immune tension [6]. Moreover, they aid gaseous exchange by secreting surface active proteins that maintain the alveolar tension in normal lung tissues. Although stationary under normal conditions, AT2 cells, which have differentiation potential, self-renew and differentiate into type 1 alveolar epithelial cells (AT1) to repair and maintain the integrity of the alveoli during lung diseases [7]. Moreover, AT2 cells inhibit the activation of inflammatory cells and suppress inflammatory responses by secreting various antibacterial substances into the alveolar fluid [6].

Despite considerable progress in the pathophysiology and treatment of ARDS, there exists no specific biomarker, treatment target, or effective prevention strategy for it. Determining gene expression changes in AT2 during the occurrence and development of ARDSp can provide insights for developing effective clinical treatments. Thus, this study aimed to elucidate the mechanism of ARDSp through investigations on AT2.

Through small nuclear RNA sequencing (snRNA-Seq) different types of cells in a sample are distinguished and the genetic information of each cell is obtained [7,8]. In this study, snRNA-Seq was used to determine the gene expression profiles of alveolar epithelial cells in ARDSp, and identify key genes and pathways involved in the occurrence and development of ARDSp. We further provided insights for the elucidation of the mechanism, treatment targets, and specific markers of AT2 injury in ARDSp, all of which contribute to the prevention of ARDSp.

## 2 Materials and methods

### 2.1 Experimental animals and groups

This study was approved by the Medical Ethics Committee of Lianyungang Clinical College of Nanjing Medical University (No: KY20200311001, Date: March 11, 2020). All the animal experiments were performed in strict accordance with the animal ethics guidelines. A total of 12 healthy clean-grade male Sprague Dawley rats were selected. Rats were six-eight weeks old, and each weighed between 230 and 250 g. They were randomly divided into either the healthy (control group) or treatment groups (ARDSp group), each of which had six animals. An ARDSp lung injury rat model (ARDSp group) was established by inoculating their airways with lipopolysaccharide (LPS) (Sigma-Aldrich, St. Louis, MO, USA; 10 mg/kg).

### 2.2 Histopathological observation of lung

All rats were anesthetized by intraperitoneal injection of methylthiazide (8 mg/kg) and ketamine (80 mg/kg) after 36 h of modeling, and were sacrificed by cardiac puncture, and samples were collected for examination. The lung tissue, from the upper two lobes of the right lung of rats, was dehydrated with alcohol, embed with paraffin and slice, dewaxed, and stained with H&E before being observed the lung tissue damage under the light microscope.

Using an electronic balance, freshly extracted lung tissue was weighted to determine the wat weight (W), and then dried in an oven at 60 °C until there was no change in weight to determine the dry weight (D). The wet-dry weight ratio was computed using the formula W/D × 100% to assess the degree of pulmonary edema.

### 2.3 Total RNA extraction from lung tissues

After 36 h, rats were anesthetized by intraperitoneal injections of xylazine (Sigma-Aldrich, St. Louis, MO, USA; 8 mg/kg) and ketamine (Hengrui, China; 80 mg/kg). After successful anesthetization, the rats were sacrificed by cardiac puncturing and bloodletting, and sample specimens were collected. From the lung tissues, total RNA was extracted using RNA extraction kits (Thermo Fischer Scientific, Waltham, MA, USA). To ensure the samples met the requirements for subsequent sequencing, the purity and integrity of the extracted RNA samples were determined using a Nanodrop one ultra-micro spectrophotometer (Thermo Fischer Scientific, Waltham, MA, USA) and an Agilent2100 Bioanalyzer (Santa Clara, CA, USA) respectively.

### 2.4 Bulk-RNA data analysis

High-throughput sequencing of multiple RNA samples was conducted using an Illumina HiSeq sequencing platform in the paired-end sequencing mode. Skewer software (version 0.2.2) was used to dynamically remove both adapter sequences from the 3′-end of the sequencing data and low-quality fragments. FastQC software (version 0.11.5) was used to perform pre-processing data quality control. For each sample, STAR software (2.5.3a) was used to align pre-processed sequences to the reference genome, and RSEQC (version 2.6.4) was used for comparative analyses. The fragments per kilobase of exon model per million mapped fragments (FPKM) method was used to calculate the level of gene expression. FPKM counts were converted into transcripts per million (TPM) counts to analyze gene differential expression in ARDSP rats.

### 2.5 Data availability

Publicly available snRNA-Seq data was used and it included that from autopsy lung tissues of 18 COVID19 patients who were infected with ARDSp and seven healthy donors. These donors’ data and clinical information are available at Gene Expression Omnibus (GEO) under the accession number GSE171524. Total RNA-Seq data from this study are available at GEO under the accession number PRJNA875078.

### 2.6 SnRNA-seq data analysis

We normalized the original matrix in each dataset by using the ‘LogNormalize’ function in Seurat (version 4.2.0). We used ‘FindVariableFeatures’ to select the 2000 most variable genes as input data. After calculating the consolidation anchors using the ‘FindIntegrationAnchors’function, the ‘IntegrateData’ function was used to integrate multiple datasets by searching for the anchors. To integrate the data, we identified the 20 best principal components (PC) by using principal component analysis (PCA) [9]. To visualize the data, we used ‘FindNeighbors’ and ‘FindClusters’ functions to resulting PCs for the computation of nearest-neighbor graphs for clustering of cells. We then applied the uniform manifold approximation and projection (UMAP) on the resulting PCs to visualize the cells. By comparing with all remaining clusters using ‘FindConservedMarkers’, we identified the conserved markers for each cluster across different conditions and identified different cell types by known cell-specific markers [10]. Markers specific for AT2 are *SFTPB*, *SFTPC*, and *SFTPD*.

### 2.7 Analysis of differentially expressed genes (DEGs)

The DEGs of AT2 in ARDSp were identified by ‘DESeq2’ using the criteria |log2FC|>0.25 and *P* < 0.05. And ‘ggplot2’ (version 3.3.6) was used to draw volcano and heat maps to illustrate differences in gene expression levels [11].

### 2.8 Identification of hub genes in AT2

We imported DEGs into the Search Tool for the Retrieval of Interacting Genes (STRING) online database (https://cn.string-db.org/) and used them to identify proteins with a comprehensive score >0.7, which were considered statistically significant. Using the cytoNCA plug-in of Cytoscape (version 3.9.1) we identified the top 10 hub genes of AT2 in ARDSp.

### 2.9 Function enrichment analyses

The GO function enrichment and KEGG pathway enrichment analyses were used to explore the potential functions of hub genes using ‘clusterProfiler’ in R. [12].

### 2.10 Statistical analyses

All statistical analyses were performed using R (version 4.1.2). All results were expressed as the mean ± standard deviation. Shapiro- Wilk normality tests for normal distribution. For comparison between groups, the Student *t*-test was used if normal distribution was met, and the Mann-Whitney *U* test was used if not. Statistical significance was considered when p < 0.05.

## 3 Results

### 3.1 SnRNA-Seq data analyses

We obtained snRNA-Seq data, including from autopsy lung tissue of 18 ARDSp patients and seven healthy donors from the GEO database, to investigate the gene expression changes in alveolar epithelial cells of patients with ARDSp. According to the clinical information of 18 ARDSp patients, 4 of them had pre-existing lung disease, 4 of them had smoking status, and 8 of them had pre-existing diabetes. All of them have respiratory symptoms. The 18 ARDSp patients included 11 men and 7 women, all of whom were over 50 years of age.

**Fig. 1:**
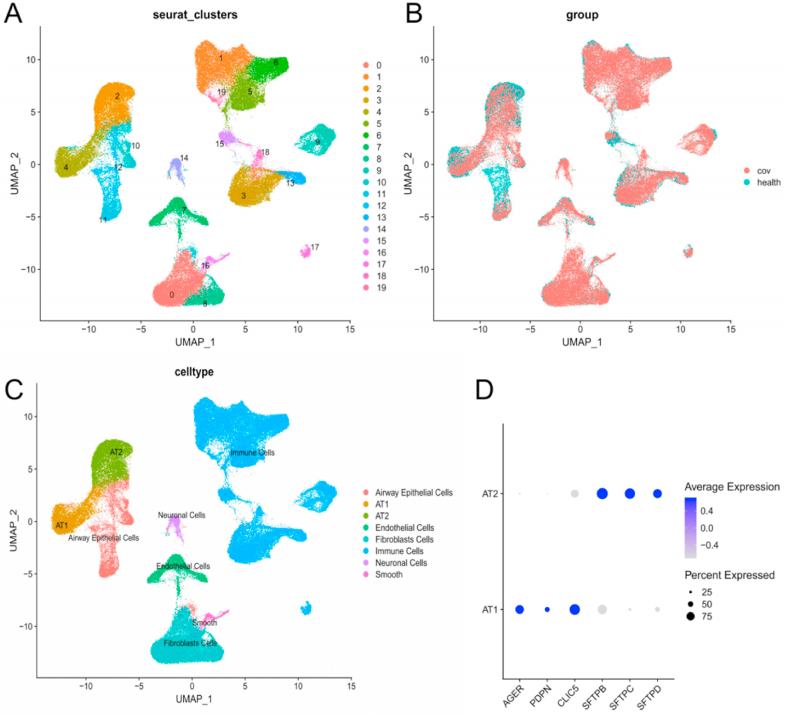
SnRNA-Seq data analysis: A) An UMAP plot displaying 20 clusters identified after an unsupervised analysis of autopsy lung tissue samples from 18 patients with ARDSp and seven healthy donors; B) An UMAP plot displaying cells from different donors in 25 autopsy lung tissue samples (18 patients with ARDSp and seven healthy donors); C) An UMAP plot displaying the major cell types (airway epithelial cells, AT1, AT2, endothelial cells, fibroblasts, immune cells, neuronal cells, Smooth muscle cells) in 20 clusters; D) A dot plot showed the expression levels of six marker genes (*AGER, PDPN, CLIC5, SFTPB, SFTPC, SFTPD*) in AT1 and AT2.

Unsupervised analysis identified 20 distinct cell clusters (Fig. 1A and B). According to the expression levels of known markers, these clusters were identified as distinct known cell types, including AT1 and AT2 (Fig. 1C). Advanced Glycosylation End-Product Specific Receptor (AGER), Podoplanin (*PDPN*), and Chloride Intracellular Channel 5 (*CLIC5*) were highly expressed in AT1. Surfactant Protein B (*SFTPB*), Surfactant Protein C (*SFTPC*), and Surfactant Protein D (*SFTPD*) were highly expressed in AT2 (Fig. 1D).

### 3.2 Patients with ARDSp had reduced cells numbers of AT1 and AT2

**Fig. 2:**
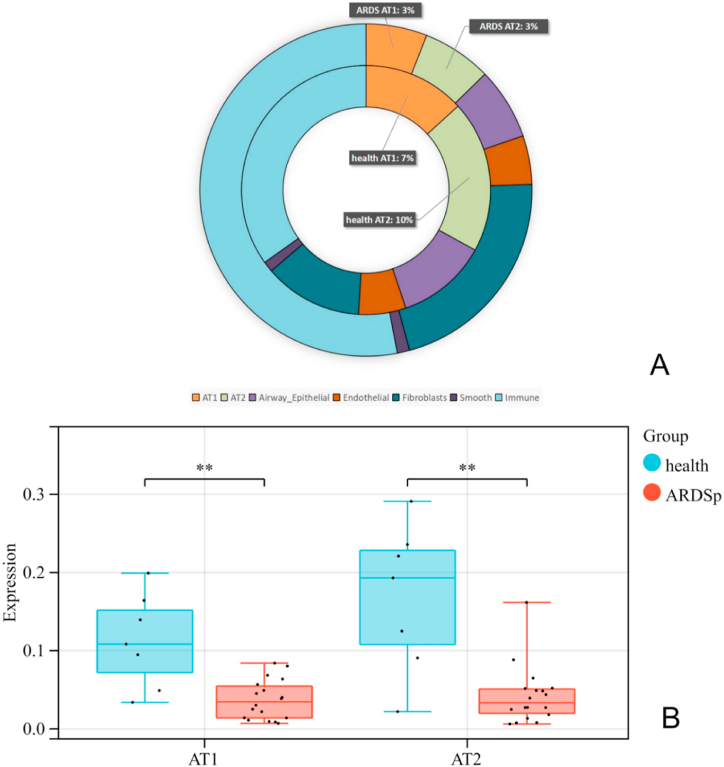
Patients with ARDSp had reduced AT1 and AT2 cell numbers. A) A circle diagram showing the proportions of various cell types. The inner and outer circles represent the proportions of various cell types in healthy donors and patients with ARDSp, respectively. B) A boxplot showing the proportion of AT1 and AT2 for both patients with ARDSp and healthy donors.

We compared the numbers of alveolar epithelial cells in lung tissue samples from patients with ARDSp to those from healthy donors. There were 4813 AT1 cells found from healthy donors, accounting for 13.12% of their total cell number. A total of 4080 AT1 cells were found from patients with ARDSp, accounting for 5.83% of their total cell number. There were 7189 AT2s found from healthy donors, accounting for 22.56% of their total cell number. A total of 4635 AT2 cells were found from patients with ARDSp, accounting for 7.03% of their total cell number (Fig. 2A). AT1 and AT2 cells were fewer in patients with ARDSp than healthy donors, and the difference in AT2 numbers was especially large between the two groups (Fig. 2B). Patients with ARDSp had more airway epithelial, endothelial, smooth, and immune cells, and fibroblasts, than healthy donors (Fig. 2A).

### 3.3 ARDSp altered gene expression in AT2

**Fig. 3:**
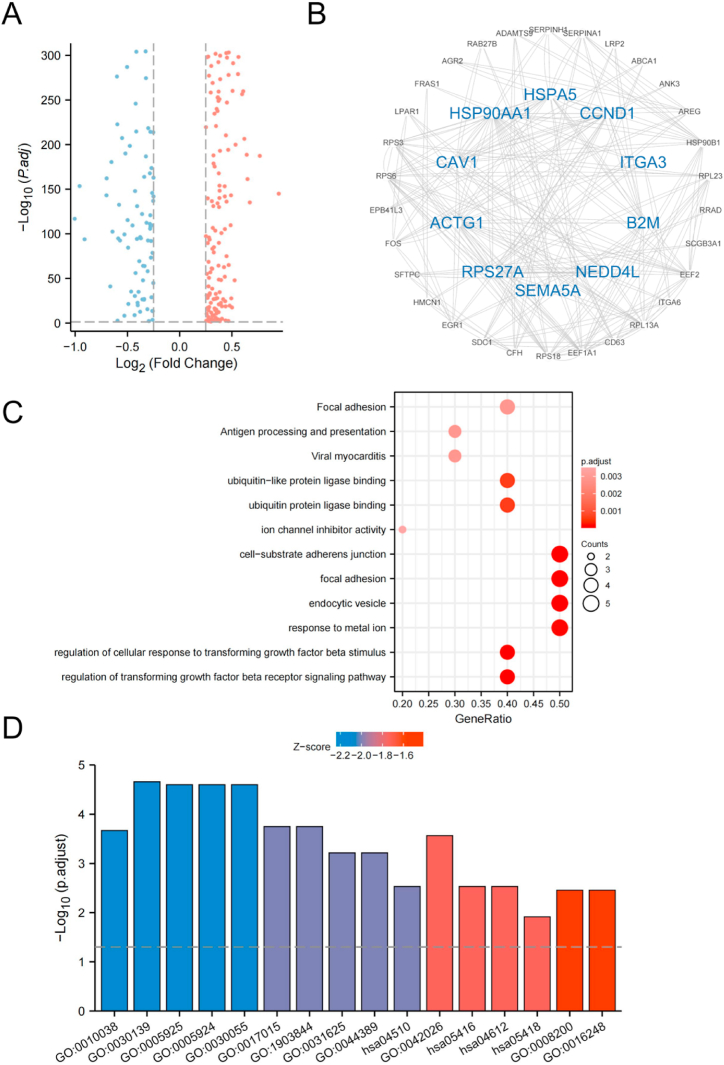
ARDSp altered gene expression in AT2. A) Volcanic map showing differentially expressed genes in AT2 of patients with ARDSp. Blue and red dots denote downregulated and upregulated genes respectively; B) The 10 predominant hub genes are in those in the center circle. Blue denotes the downregulated genes; C) A bubble plot showing the distribution of hub genes in biological processes (BP), cellular components (CC), molecular functions (MF), and KEGG pathways. The X-axis represents the gene ratio, and the y-axis represents the GO term; D) A bar chart showing the results of GO and KEGG function enrichment in combination with Log2FC of hub genes.

We determined changes in AT2 gene expression due to ARDSp. AT2 from ARDSp patients had a total of 289 DEGs, including 190 upregulated genes and 99 downregulated genes, when compared to those from healthy donors (Fig. 3A).

A protein-protein interaction (PPI) network was used to determine the interactions between these DEGs. From the 224 node proteins, 1165 interacting edges (the expected number of edges was 478) were identified, with an average node degree of 10.4, an average local clustering coefficient of 0.463, and an enrichment of *P* < 10e-16. Using the Cytoscape plug-in cytoNCA, we then identified the 10 predominant hub genes: Ribosomal Protein S27a (*RPS27A)*, Actin Gamma 1 (*ACTG1)*, Caveolin 1 (*CAV1)*, Heat Shock Protein 90 Alpha Family Class A Member 1 (*HSP90AA1)*, Heat Shock Protein Family A Member 5 (*HSPA5)*, Cyclin D1 (*CCND1),* Integrin Subunit Alpha 3 (*ITGA3)*, Beta-2-Microglobulin (*B2M)*, NEDD4 Like E3 Ubiquitin Protein Ligase (*NEDD4L)*, and Semaphorin 5A (*SEMA5A)* (Fig. 3B, Table 1).

**Table 1:** Log2FC of Hub genes.

Gene Name	p.value	Log2FC
RPS27A	<0.001	0.378612112
ACTG1	2.56E-107	0.317258101
CAV1	1.7E-19	0.404572566
HSP90AA1	1.19E-145	0.948331085
HSPA5	5.86E-149	0.382383461
CCND1	<0.001	0.403406923
ITGA3	0.006820412	0.279393895
B2M	<0.001	0.63081069
NEDD4L	3.93E-17	0.333848173
SEMA5A	0.007729882	0.384345898

Using the criteria *P* < 0.05 and q value < 0.2, GO function and KEGG pathway analyses of the 10 hub genes showed their enrichment in 367 biological processes (BPs), 32 cellular components (CCs), 32 molecular functions (MFs), and 12 signaling pathways (Fig. 3C and D). The BP in which the hub genes participated mainly included regulation of cellular response to both the transforming growth factor beta stimulus and metal ions. The hub genes were mainly enriched in the endocytic vesicle, focal adhesion, and cell-substrate junction CCs. The hub genes were mainly enriched in the MFs of ubiquitin protein ligase binding, ion channel inhibitor, and channel inhibitor activities (Fig. 3C and D, Table 2).

**Table 2:** GO and KEGG enrichment of Hub genes.

ONTOLOGY	ID	Description	GeneRatio	BgRatio	p.adjust	qvalue
BP	GO:0017015	regulation of transforming growth factor beta receptor signaling pathway	4/10	120/18670	1.78e-04	7.42e-05
BP	GO:1903844	regulation of cellular response to transforming growth factor beta stimulus	4/10	122/18670	1.78e-04	7.42e-05
BP	GO:0010038	response to metal ion	5/10	364/18670	2.14e-04	8.92e-05
BP	GO:0042026	protein refolding	3/10	40/18670	2.72e-04	1.14e-04
BP	GO:0007179	transforming growth factor beta receptor signaling pathway	4/10	199/18670	5.04e-04	2.10e-04
CC	GO:0030139	endocytic vesicle	5/10	303/19717	2.20e-05	1.03e-05
CC	GO:0005925	focal adhesion	5/10	405/19717	2.52e-05	1.18e-05
CC	GO:0005924	cell-substrate adherens junction	5/10	408/19717	2.52e-05	1.18e-05
CC	GO:0030055	cell-substrate junction	5/10	412/19717	2.52e-05	1.18e-05
CC	GO:0030666	endocytic vesicle membrane	3/10	167/19717	0.002	7.22e-04
MF	GO:0031625	ubiquitin protein ligase binding	4/10	290/17697	6.09e-04	2.38e-04
MF	GO:0044389	ubiquitin-like protein ligase binding	4/10	308/17697	6.09e-04	2.38e-04
MF	GO:0008200	ion channel inhibitor activity	2/10	37/17697	0.003	0.001
MF	GO:0016248	channel inhibitor activity	2/10	38/17697	0.003	0.001
MF	GO:0015459	potassium channel regulator activity	2/10	52/17697	0.005	0.002
KEGG	hsa05416	Viral myocarditis	3/10	60/8076	0.003	0.002
KEGG	hsa04510	Focal adhesion	4/10	201/8076	0.003	0.002
KEGG	hsa04612	Antigen processing and presentation	3/10	78/8076	0.003	0.002
KEGG	hsa05418	Fluid shear stress and atherosclerosis	3/10	139/8076	0.012	0.008
KEGG	hsa04530	Tight junction	3/10	169/8076	0.017	0.011

### 3.4 Validation of hub genes via total RNA sequencing

**Fig. 4:**
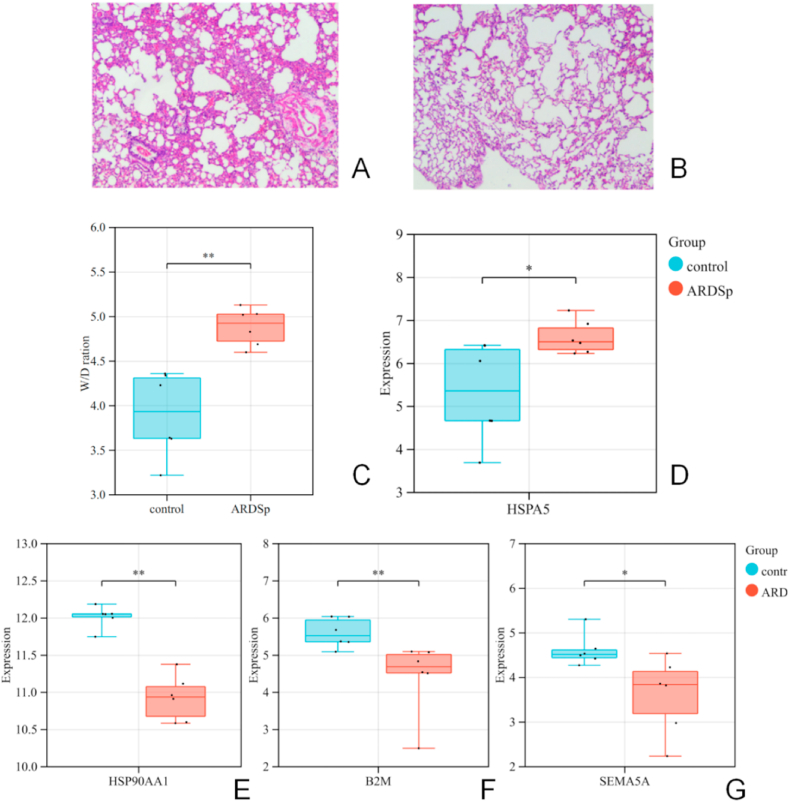
Validation of hub genes by total RNA sequencing: A) Pathological image of the lung tissue in the sepsis-induced ARDS lung injury model group (magnification 40); B) Pathological image of the lung tissue in the control group (magnification 40); C) The histogram shows theW/D ratio in the control group and the ARDSp group; D) The expression of HSPA5 was upregulated in the ARDSp group. E–G) The expression of HSP90AA1, B2M, and SEMA5A was downregulated in the ARDSp group.

We constructed an ARDSp rat model through induction via LPS airway instillation and after 36 h. The pathological changes in the rat lung tissue were evaluated. Compared with the control group, the lung tissue in the LPS group showed more serious pathological damage (Fig. 4A and B).

The wet-dry weight ratio (W/D ratio) also showed more serious degree of pulmonary edema (Fig. 4C). Total RNA was extracted from the lung tissues. Lung RNA was then sequenced via Illumina Hiseq platforms and total RNA sequencing data was used to further analysis. 4471 DEGs were identified in validata data, including 1822 upregulated genes and 2649 downregulated genes. After verified the expression levels of hub genes in validata data. *HSPA5* was similarly differentially expressed like in cells from patients with ARDSp (Fig. 4D). Although *HSP90AA1*, *B2M,* and *SEMA5A* were also differentially expressed, it was downregulated (Fig. 4E–G).

## 4 Discussion

This study aimed to investigate hub genes and signaling pathways of AT2 injury in ARDSp. We specifically determined the changes in gene expression in AT2 to explore the mechanism of AT2 injury during the occurrence and development of ARDSp and aid the elucidation of targets for its prevention and treatment.

After defining cell clusters such as AT1, AT2, airway epithelial cells, and fibroblasts, we determined the changes in cell numbers for each cluster. Those for AT1 and AT2 had significantly decreased (Fig. 2B), corroborating the pathological changes of diffuse alveolar injury in ARDSp.

In the PPI interaction analysis of 245 DEGs, we identified a total of 224 node proteins, 1165 interaction edges, and an average node degree of 10.4. This strong interaction and high enrichment suggested that the differentially expressed proteome conferred biological activity during the development of ARDSp — there was a clear correlation in function.

The GO function and KEGG pathway analyses of 10 hub genes showed that regulation of the transforming growth factor beta receptor signaling pathway, cellular response to transforming growth factor beta (TGF-β) stimulus, and responses to metal ions played an important role in AT2 injury (Fig. 3C, Table 1).

Human ARDSp presents at three stages: exudative, fibro-proliferative and fibrotic. Considering that all 18 ARDSp patients in our study died of respiratory failure, we focused on the possible mechanisms in exudative.

It is worth noting that TGF-β associated signaling pathways, which were enriched with CAV1, HSPA5, ITGA3, and RPS27A, are closely related to the occurrence and development of ARDSp. Relatedly, TGF-β associated signaling pathways can participate in and regulate the proliferation and differentiation of AT2. TGF-β promoted the proliferation of AT2 in injured alveolar epithelial cells. After the replacement of injured cells, TGF-β expression was inhibited, thus inducing AT2 cell cycle arrest, which promoted AT2 differentiation. Meanwhile, higher than normal physiological levels of TGF-β not only led to the failure of AT2 to differentiate after completing proliferation but also induced epithelial-mesenchymal. The function of TGF-β in the progression of lung fibrosis has been reported. It has been reported that curdione attenuates bleomycin-stimulated lung fibrosis by inhibiting TGF-β-related fibroblast to myofibroblast differentiation [13]. Moreover, the alveolar epithelial cell is the prime target during injury where this cell undergo epithelial-mesenchymal transition (EMT). TGF-β is one of the most important factors inducing EMT. It has been reported that biochanin-A suppresses lung fibrosis by repressing the TGF-β-mediated collagen deposition, myofibroblasts differentiation, and EMT [14]. Epithelial injury and pulmonary fibrosis are both important mechanisms in the development of ARDS. Understanding the role of TGF-β will help us improve our understanding of the development process of ARDS. At the same time, we found that ITGA3 is closely related to EMT. On the one hand, several studies have found that EMT plays an important role in ARDS [15,16], on the other hand, ITGA3 has been confirmed to participate in the development process of tumors by participating in EMT in a variety of tumors [17,18]. We believe that it is meaningful to conduct subsequent experiments to specifically explore whether ITGA3 can play a role in the occurrence and development of ARDSp by participating in EMT.

Analysis of total RNA sequencing data from lung tissues of the ARDSp rat model verified that expression levels of *HSPA5* was ipregulated in the ARDSp group (Fig. 4D). At present, the relationship between HSPA5 and ARDS has not been directly proved by research. However, studies have proved that HSPA5 is closely related to mitochondrial stress and iron death [19], and the latter is related to the development of ARDS [20]. Mitochondria are the main sites where cells produce reactive oxygen species. Under normal conditions, intracellular ROS can be used as intracellular signal molecules to regulate normal physiological functions of the body. When the tissue is damaged, the reactive oxygen species in the mitochondria increases greatly, which damages the mitochondria and leads to mitochondrial dysfunction. Endoplasmic reticulum stress is related to pulmonary fibrosis. Pro-fibrotic effects of ER stress may be transduced through several different cell types in the lungs, including alveolar epithelial cells, fibroblasts, and macrophages [21]. At the same time, it is reported in some articles that inhibition of Heme oxygenase-1 (HO-1), which also plays an important role in ferroptosis, can inhibit ER stress in the lung, thus reducing ARDS. Further experiments should be carried out to verify whether the development of ARDS can be inhibited by inhibiting the expression of HSPA5.

This study had limitations. For snRNA-Seq data, it is unclear at what stage of ARDSp the patient was in or how severely inflamed the lung tissue was during sampling. Thus, snRNA-Seq data for lung samples at either different ARDSp stages or degrees of inflammation would have enhanced the accuracy of the analysis. The snRNA data analyzed were only from COVID19 patients with ARDSp. Considering that only one subtype of ARDSp is analyzed and there are unique aspects of COVID19 lung, the conclusion of the experiment may not be perfect, and follow-up experiments need to be carried out to verify other ARDSp subtypes.

In this experiment, the bulk RNA-Seq data of male rats with ARDSp induced by LPS airway infusion were used for validation. It has not been verified in other rat models with different ARDSp subtypes, nor has the effect of gender differences been discussed. Species differences may have led to variations, it would be more convincing if human lung tissue with different subtypes of ARDSp could be used for verification. Furthermore, the limitations of conventional bulk RNA-Seq may also have influenced the findings.

## 5 Conclusion

In conclusion, 255 DEGs associated with injured AT2 in ARDSp were identified. Ten hub genes (*RPS27A, ACTG1, CAV1, HSP90AA1, HSPA5, CCND1, ITGA3, B2M, NEDD4L, SEMA5A*) were identified, three of which, *HSPA5* was experimentally verified via total RNA-Seq. These findings increase insights into ARDSp and might be aid treatment of ARDSp.

## Fund projects

The Social Development Project of Jiangsu Provincial Department of Science and Technology (BE2020670); General Project of Jiangsu Provincial Health Commission (H2019109); The Social Development Project of Lianyungang Science and Technology (SF2117) Joint funding.

## Author contribution statement

Haoran Chen: Conceived and designed the experiments; Performed the experiments; Analyzed and interpreted the data; Contributed reagents, materials, analysis tools or data; Wrote the paper.

Jinqiu Ding: Haoyue Xue: Xinyi Tang: Yao Yan: Performed the experiments.

Yongpeng Xie: Conceived and designed the experiments; Performed the experiments; Contributed reagents, materials, analysis tools or data.

## Data availability statement

Data associated with this study has been deposited at [ScRNA-Seq, Total RNA-Seq] under the accession number [GSE171524, PRJNA875078].

## Declaration of competing interest

The authors declare that they have no known competing financial interests or personal relationships that could have appeared to influence the work reported in this paper.

## Abbreviations

ARDSp: Pulmonary Acute Respiratory Distress Syndrome
snRNA-Seq: Single Nuclear RNA Sequencing
AT2: Type 2 Alveolar epithelial cells
DEGs: Differentially Expressed Genes
STRING: Search Tool for the Retrieval of Interacting Genes
LPS: Lipopolysaccharide
GO: Gene Ontology
KEGG: Kyoto Encyclopedia of Genes and Genomes
ARDS: Acute Respiratory Distress Syndrome
ICU: Intensive Care Unit
AT1: Type 1 Alveolar epithelial cells
FKPM: The Fragments Per Kilobase of Exon Model Per Million Mapped Fragments
TPM: Transcripts Per Million
GEO: Gene Expression Omnibus
PC: Principal Components
PCA: Principal Component Analysis
UMAP: Uniform Manifold Approximation and Projection
AGER: Advanced Glycosylation End-Product Specific Receptor
PDPN: Podoplanin
CLIC5: Chloride Intracellular Channel 5
SFTPB: Surfactant Protein B
SFTPC: Surfactant Protein C
SFTPD: Surfactant Protein D
RPS27A: Ribosomal Protein S27a
ACTG1: Actin Gamma 1
CAV1: Caveolin 1
HSP90AA: 1Heat Shock Protein 90 Alpha Family Class A Member 1
HSPA5: Heat Shock Protein Family A Member 5
CCND1: Cyclin D1
ITGA3: Integrin Subunit Alpha 3
B2M: Beta-2-Microglobulin
NEDD4L: NEDD4 Like E3 Ubiquitin Protein Ligase
SEMA5A: Semaphorin 5A
BPs: Biological Processes
CCs: Cellular Components
MFs: Molecular Functions
TGF-β: Transforming Growth Factor Beta
PPI: Protein-Protein Interaction
EMT: Epithelial-Mesenchymal Transition
NO: Nitric Oxide
HO-1: Heme Oxygenase-1
